# Role of the HGF/c-MET tyrosine kinase inhibitors in metastasic melanoma

**DOI:** 10.1186/s12943-018-0795-z

**Published:** 2018-02-19

**Authors:** Lucia Demkova, Lucia Kucerova

**Affiliations:** Laboratory of Molecular Oncology, Cancer Research Institute, Biomedical Research Center of the Slovak Academy of Sciences, Dubravska cesta 9, 845 05 Bratislava, Slovakia

**Keywords:** Metastasis, Malignant melanoma, Hepatocyte growth factor, C-met receptor, Tyrosine kinase inhibitors, A375 Human malignant melanoma cell line.

## Abstract

Metastatic disease in a cancer patient still remains a therapeutic challenge. Metastatic process involves many steps, during which malignant cells succeed to activate cellular pathways promoting survival in hostile environment, engraftment and growth at the distant site from the primary tumor. Melanoma is known for its high propensity to produce metastases even at the early stages of the disease. Here we summarize the most important molecular mechanisms which were associated with the melanoma metastasis. Then, we specifically focus on the signaling pathway mediated by hepatocyte growth factor (HGF) and its receptor c-Met, which play an important role during physiological processes and were been associated with tumorigenesis. We also focus on the effect of the small molecule inhibitors of the tyrosine kinase domain of the c-Met receptor and its effects on properties of melanoma cell. We summarize recent studies, which involved inhibition of the HGF/c-Met signaling in order to decrease melanoma growth and metastatic capacity.

## Background

Metastatic dissemination still represents a major problem in the treatment of cancer and it still remains the most common cause of death in cancer patients. Metastasis is the result of complex multilevel processes. Malignant abnormal cells grow beyond their usual borders, invade adjacent parts surrounding tumor and spread to other organs. Accumulation of alterations in tumor cells leads to appearance of metastatic cancer cells. They become invasive after detaching from the primary tumor, because they acquire ability to penetrate to the bloodstream or the lymphatic system, to grow and thrive in their new location, and to induce angiogenesis. There are some general principles in the metastatic cascade among the various tumor types, although this complex process still remains to be fully understood [1].

Malignant melanoma arises from melanocytic cells and primarily involves a skin, less frequently an eye (uvea, conjunctiva and ciliary body), meninges and mucosal surfaces [2]. Even though it is considered to be human tumor with the most prominent immunogenic response, malignant melanoma represents one of the most insidious cancer for its ability to rapidly disseminate and induce metastasis [3]. Exogenous risk factors such as an exposure to ultraviolet light, increase melanoma incidence, as well as skin damage with burns, frostbite and damage after radiotherapy, although the mechanism of the latter is not always clear. Compromised immune system as a result of chemotherapy, an organ transplant and diseases such as HIV/AIDS or lymphoma also increase the risk of melanoma [4, 5].

Melanomas metastasize either by direct dissemination, by the lymphatic or the haematogenous route [2]. In melanoma patients, thorough examination and detailed staging including high-resolution imaging techniques, such as USG, PET, CT or magnetic resonance imaging is necessary to unravel distant metastases [6, 7]. Primary treatment of the melanoma is surgical excision and its extirpation with the surrounding subcutaneous tissue with skin safety margin ‘in bloc’ [6]. In the case of isolated locoregional lymph node (LN) metastases, surgical removal of the tumor-bearing LN alone is insufficient. Non-resecable in-transit metastases may be controlled by radiotherapy alone. Radiotherapy effectively palliates pain caused by bone metastases. Melanoma has a marked propensity to metastasize to the brain. With radiation therapy, the neurologic deficits may be improved in 50–75% of cases, an effect which is usually associated with an overall improvement in health [2, 6, 7].

High risk of microscopic metastases in melanoma patients is handled by using adjuvant therapies in order to delay the recurrence of disease. However, a number of controlled trials with adjuvant chemotherapy in stage II and III patients did not demonstrate any therapeutic advantage. Adjuvant immunotherapy by Interferon (IFN)-α is the treatment of melanoma which has shown a significant improvement of disease-free survival, and also impact on overall survival, albeit with significant toxicity. Ongoing clinical trials test the efficiency of immune system-activating monoclonal antibodies Ipilimumab and Pembrolizumab in melanoma treatment [2, 8, 9]. For the patients, who are candidates for systemic medical treatment, molecular analysis of distant or regional metastasis of the primary tumor is performed for BRAF V600 mutations, NRAS mutations and NF1 mutations. Based on the detection of the specific mutation, the BRAF/MEK inhibitors are used of in adjuvant targeted therapy. There are two prospectively randomized trials on either Vemurafenib alone (BRIM8) or the combination of Dabrafenib and Trametinib (COMBIAD) [2, 9].

In general, there is still limited number of options for systemic therapy in patients with inoperable regional and distant metastases. Most significant effect on tumor responses was achieved by immunotherapy or targeted therapy using small-molecule drugs and monoclonal antibodies so far. Better understanding of the complex metastatic cascade may unravel novel targets to limit the spread of malignant cells. One of the important signaling pathways which was implicated to play role in many cancers including metastatic spread is signaling by hepatocyte growth factor (HGF) via its cognate receptor c-Met with tyrosine kinase activity. Here we focus on the small-molecule inhibitors of this signaling pathway and its effect on metastatic melanoma.

### Mechanisms of metastatic dissemination

Metastatic spread is a multi-step process releasing tumor cells from a primary lesion to a disparate organ or organs within the body. Tumor cells change their characteristics throughout the process, which enable them to proliferate and migrate, invade surrounding tissue, intravasate through the basement membrane into blood or lymphatic vessels, survive during circulation through the blood or lymphatic system, stay at distant sites, extravasate into a new environment tissue, and proliferate by inducing angiogenesis [10–12].

The classical metastatic cascade starts from a primary, epithelial, neoplastic lesion and includes: (1) epithelial – mesenchymal transition (EMT) with a breach of the basement membrane barrier; (2) dissociation of tumor cells from the primary tumor mass, (3) invasion of the neighboring tissue, (4) intravasation into pre-existing and newly formed blood and lymph vessels, (5) transport through the vessels, (6) extravasation from the vessels, (7) establishment of circulating tumor cells (CTCs) and the disseminated cells at a secondary anatomical site, where they can stay dormant for a prolonged period of time, (8) metastatic outgrowth of micrometastases and macrometastases creating clinically detectable secondary tumors and neoplastic process [13]. Each of these phases is composed of multiple steps. The pre-colonization phase of metastasis comprises a series of events that occur on a timescale of minutes to hours. Local invasion from the primary tumor is followed by the intravasation of tumor cells into the vasculature. The cancer cells then enter the circulatory system as single cells or clusters that are coated with platelets. Circulatory patterns, which move blood through the lungs and then on to other organs, and the differing structure of the capillary walls in each organ influence the dissemination of CTCs. On their arrest in capillaries at distant sites, the cancer cells extravasate into the parenchyma of target organs to commence colonization. Colonization can be divided into many steps that occur on a timescale of years. After extravasation, colonizing cancer cells must develop resistance to immunity and other host-tissue defenses to survive. Settlement in supportive niches enables them to survive and retain their stem-like tumor-initiating capacity. The cancer cells then enter a latent state as single cells or micrometastases. During latency, which can last from months to decades, disseminated cells must achieve long-term survival. They might also acquire traits that are required to overtake host tissue. When the cancer cells break out of latency, they reinitiate overt outgrowth and overtake the local tissue microenvironment. Therapeutic treatment can partially eliminate clinically manifest metastases. However, under therapy-induced stress, cancer cells and non-neoplastic stromal cells mobilize survival signals that nurture the residual disease until minority drug-resistant clones emerge to lead the outgrowth of a drug-resistant tumor. Different host-tissue microenvironments select for cancer cells with distinct metastatic traits, which gives rise to organ-specific populations of metastatic cells [14].

Tumor cells employ different strategies of migration to invade into the stroma and proceed toward the blood or lymph stream: single-cell migration and collective migration [12, 15]. The loss of the epithelial cell-to-cell adhesion molecule E-cadherin, the major component of epithelial adherence junctions, is needed upon the migration and invasion induction [15, 16]. Mesenchymal single-cell invasion is characteristic by the spindle-shaped morphology of the cancer cells (mesenchymal phenotype), and the expression of proteases and integrins. Characteristics for the amoeboid invasion are: weak interactions with the extracellular matrix and protease independency [12, 17]. Cells migrating with low adhesion force or high actomyosin-mediated contractility adopt morphologically spherical shapes. The amoeboid and mesenchymal types of migration are mutually interchangeable [18]. When individual cells (both mesenchymal and amoeboid) move one after another using the same path within the tissue, it is called as multicellular streaming [15, 18]. Collectively migrating cells retain their cell–to-cell junctions through continuous expression of adhesion molecules. This type of invasion is protease-dependent. They migrate as sheets, strands, tubes or clusters and remain either connected to the primary tumor (coordinated invasion) or move as detached cell groups or clusters (cohort migration) [12, 17]. Collectively migrating cells can display mesenchymal or epithelial phenotypes, and the phenotypes may differ between ‘leader’ and ‘follower’ cells in some cases [15].

The microenvironment at the invasive edge of tumors is quite different than that of the tumor core. Tumor microenvironment has diverse capacities to induce both beneficial and adverse consequences for tumorigenesis and the microenvironment supports metastatic dissemination and colonization at secondary sites. Macrophages, platelets, and mesenchymal stem cells contribute to the EMT at primary sites, allowing for tumor cells to separate from neighboring epithelial cell-to-cell contacts and acquire an invasive phenotype. One major mediator of this event is transforming growth factor-beta (TGF-β), which is secreted by the tumor stroma and participates in a paracrine signaling loop with tumor cells [19]. A large number of growth factors and their activated signal transduction pathways are known to provoke the loss of E-cadherin function and induce cancer cell migration and invasion [16].

Tumor-associated macrophages (TAMs), cancer-associated fibroblasts (CAFs) and myeloid progenitor cells also tend to cluster at the invasive edge of the primary tumor, where they play an immunosuppressive role by interfering with dendritic cell differentiation. During intravasation of tumor cells into circulation, intravital imaging studies have shown that macrophages are localized to perivascular areas within tumors, where they help tumor cells to traverse vessel barriers [20]. In the circulation, platelets and components of the coagulation system support tumor cell survival by protecting them from cytotoxic immune cell recognition. Platelets escort tumor cells in circulation to the site of extravasation, where they bind to areas of vascular retraction and help tumor cells to exit circulation into secondary organs. At secondary sites such as the lung, fibroblasts upregulate fibronectin, which serves as a docking site for hematopoietic progenitor cells and the subsequent arrival of tumor cells. Immunosuppressive cell types, such as myeloid-derived suppressor cells and natural killer cells (NK cells), also populate pre-metastatic niches where they help to direct metastatic dissemination by creating a niche permissive to tumor colonization. Recent studies have demonstrated that primary and secondary sites can communicate through exosomes, shed not only by the primary tumor cells, but also by the immune and stromal cells such as NK cells, CAFs and dendritic cells [19].

Factors contained in exosomes have the capacity to direct organ tropism, modulate immune evasion, support mesenchymal-to-epithelial transition (MET), and they are predictive of metastasis and patient outcome. Tumor exosomes could also facilitate organ-specific metastatic behavior by preparing pre-metastatic niches [19, 21].

Stephen Paget more than 120 years ago (in 1889), proposed a hypothesis of “seed and soil” to describe metastatic outgrowth. Paget observed that primary tumor cells of a given type of cancer preferentially metastasized to one or more particular distant organ sites, detectable metastases only developed at those sites (“soils”) where the tumor cells (“seeds”) were adapted for survival and proliferation [21]. However, recent research indicates that the primary tumor may determine organotropic metastases by inducing the formation of pre-metastatic niches. Specifically, exosome vesicles secreted by tumors have been shown to home to pre-metastatic sites, where they activate pro-metastatic processes such as angiogenesis, and modify the immune contexture, so as to foster a favorable microenvironment for secondary tumor [22]. The most common sites, where the primary tumors tend to spread, are the bone, liver, and lung. However, melanoma cells preferentially metastasize to brain, liver, lung, skin, muscle and bones.

### Molecular mechanisms of metastasis

The genes, which allow transformed cells to invade the surrounding tissue and attract a supportive stroma, can be defined as metastasis initiation genes and could promote cell motility, EMT, extracellular matrix degradation, bone marrow progenitor mobilization, angiogenesis or evasion of the immune system. Other determinants of invasion are components and modulators of the HGF/c-Met pathway, such as metadherin in breast cancer and the metastasis-associated in colon cancer 1 (*MACC1*) gene in colorectal carcinoma. The expression of these metastasis initiation genes and their targets predicts a poor prognosis in particular types of cancer [11]. Many of the molecular players involved in early invasion events have been mechanistically linked to metastasis in experimental and clinical settings; among those, GTPases or their activators/inhibitors (Tiam-1 - T-cell lymphoma invasion and metastasis-inducing protein 1, Rho-C - Ras homolog gene family, member C), and receptor tyrosine kinase (RTK) upstream of Rho GTPases as hepatocyte growth factor receptor (HGFR/c-Met) or tyrosine kinase receptor (Trk-A) [22]. Isoform TrkA-III promotes angiogenesis and has oncogenic activity when overexpressed [23].

The key signaling pathways and molecules inducing EMT include receptor tyrosine kinases (RTKs), TGF-β superfamily, WNT, NOTCH, hedgehog pathway and NF-κB [13]. The PI3K/AKT pathway is an important regulator of cell cycle progression; and therefore it is a frequent contributor to cellular transformation, when normal function is compromised via genetic or epigenetic modifications. Conventional activation of the pathway is initiated at the cell surface by the phosphorylation of RTKs in response to mitogen stimulation [10]. The regulation of diverse transcription factors, receptors for growth factors (including FGFR2b, FGFR2c, EGFR and HER2), and the activation of Akt are other elements in the reversion of MET [24].

Signaling events leading to EMT activate mesenchymal state in the cancer cells, which was associated with increased frequency of cancer stem cells (CSCs) sometimes also termed tumor-initiating cells [25]. These tumorigenic cells are like adult or embryonic stem cells in their ability to self-renew and give rise to a diversity of cells that differentiate and after a finite number of divisions, eventually succumb to programmed cell death. CSCs differ from the adult stem cells in that their division results in tumor initiation and growth [26]. Recently, it has been suggested that melanomas may be derived from transformed melanocytic stem cells, melanocyte progenitors, or de-differentiated mature melanocytes [27].

Melanoma stem cells have been identified in both primary tumors and cell lines. There are several key stem cells markers specified for malignant melanoma: CD20, CD133, ABCB5, CD271 and ALDH1A [28]. Recently identified melanoma stem cell markers include JARID1B (jumonji, AT-rich interactive domain 1B), ABCB5 (ATP-binding cassette subfamily B (MDR/TAP) member 5), ABCG2 (ATP-binding cassette subfamily G member 2), and MDR1 (multi-drug resistance 1) [27]. These JARID1B-positive melanoma cells gave rise to a highly proliferative progeny, and knockdown of JARID1B led to accelerated tumor growth, which was followed by exhaustion. Perhaps then, this small population of JARID1B-positive was required for continuous tumor growth. However, expression of JARID1B was not consistent, and did not follow a hierarchical cancer stem cell model: even JARID1B-negative cells could become positive and even single melanoma cells were tumorigenic in xenografts [29]. One key molecular marker to target is ABCB5. Importantly, ABCB5 is not only a biomarker of melanoma stem cells, but also provides a mechanism for chemoresistance. Several potential therapies against ABCB5 have been explored, including monoclonal antibodies [30]. In the study by Fusi et al. authors reported that melanoma cells in peripheral blood expressed stem cell-associated markers Nestin and CD133 [31]. Higher expression of Nestin by CTCs might represent an index of poor prognosis. Nestin expression is associated with cell migration and metastasis in prostate cancer, and tumor progression and deceased survival in melanoma. Nestin and SOX2 are embryologic stem cell transcription factors that bind an enhancer region on the nestin gene, and they are preferentially co-expressed in metastatic melanomas when compared with nevi or primary melanomas. Moreover, SOX2-positive melanoma cells tend to be more spindle-shaped and have more peripheral nestin pattern, which may represent a motile, more mesenchymal phenotype [26]. Melanoma cell survival in the bloodstream can be attributed to mechanisms that ensure evasion from attack by natural killer (NK)-cells, the most potent mode of host defense against cancers. One such mechanism, which provides immune privilege and prevents NK-cell-mediated cytotoxicity, is the intracellular localization within melanoma cells of the ligand that typically activates NKD2D receptors on NK-cells [31].

Transmembrane RTKs are composed of a single transmembrane domain that separates the intracellular tyrosine kinase region from the extracellular part. RTKs contain tyrosine kinase and they have a high affinity for polypeptides, such as growth factors, cytokines and some hormones (especially insulin). RTKs play an important role in the physiological developmental processes and the development of many cancers [32]. Human RTKs containing 20 subfamilies including epidermal growth factor receptor (EGFR), vascular endothelial growth factor receptor (VEGFR), platelet-derived growth factor receptors (PDGF-R), fibroblast growth factor receptors (FGFR), insulin-like growth factor 1 receptor (IGF-1R), and hepatocyte growth factor receptor (c-Met or HGFR). They have shown a substantial level of crosstalk bringing another level of complexity into the signaling events [33, 34]. As the essential components of signal transduction pathways, which mediate cell-to-cell communication, these single-pass transmembrane receptors play key role in processes such as cellular growth, differentiation, metabolism and motility. Dysregulation of certain RTKs has been implicated in the development and progression of many types of cancer [35, 36]. Expression of the receptor c-Met and its only known ligand HGF, has been observed in tumor biopsies of solid tumors, and the c-Met signaling has been documented in a wide range of human malignancies (for example in brain tumors and non-small cell lung cancer; medulloblastomas; gastric and esophageal carcinomas) [36–39].

In various solid tumors, including gastric, breast, thyroid and hepatocellular carcinomas, the HGF/c-Met pathway was detected as a critical in cancer development [14]. In a recent paper by Bendinelli et al. it was demonstrated for the first time that the interaction between HGF and epigenetic mechanisms controlling gene expression is important for the metastatic phenotype. Their data indicated the importance of targeting the tumor microenvironment by blocking epigenetic mechanisms, which control critical events for colonization such as HGF/c-Met axis as a potential therapy of bone metastasis [40].

### HGF and c-met signaling

The c-Met receptor and the HGF are found in a many tissues and organs, but their expression is usually restricted to the cells of epithelial and mesenchymal origins. Mouse genetic studies discovered that both c-Met and HGF are important in embryonic development, organ morphogenesis and cell motility. In adults, their activities are more restricted, predominantly in tissue regeneration and damage repair [41–44].

HGF acts on a wide variety of epithelial cells as a mitogen (stimulation of cell growth), a motogen (stimulation of cell motility), and a morphogen (induction of multicellular tissue-like structure). Due to these functions HGF is considered a key molecule for the construction of normal tissue structure during embryogenesis, organogenesis, and organ regeneration [45]. HGF (also known as scatter factor SF) is a large, multi-domain protein that is similar to plasminogen, a circulating proenzyme, the active form of which is responsible for the lysis of blood clots [41]. The gene encoding HGF spans approximately 70 kb on chromosome 7q21.1 and consists of 18 exons and 17 introns [46]. Mature HGF is a heterodimer, consisting of a 69 kDa alpha- and a 34 kDa beta-chain held together by a single disulfide bond [47]. HGF is produced mainly in the liver. Kupffer cells play a stimulatory role in liver regeneration by enhancing HGF expression [48].

Its cognate receptor c-Met is a protein that is encoded in humans by the *MET* gene located on chromosome 7 (bands q21–q31) and consists of 21 exons separated by 20 introns [49]. The extracellular domain of the c-Met presents two subunits, linked by a disulfide bond, which form the mature receptor for HGF. In the wild-type cells, the primary c-Met transcript produces a 150 kDa polypeptide that is partially glycosylated to produce a 170 kDa precursor protein. This 170 kDa precursor is further glycosylated and then cleaved into a 50 kDa-chain and a 140 kDa-chain which are linked via disulfide bonds [47]. The intracellular domain is constituted of a juxta membrane domain, involved in the receptor down-regulation, a tyrosine kinase domain, involved in signal transduction, and a C-terminal regulatory tail [50]. The human c-Met receptor gene is a prototypic member of the subclass IV receptor tyrosine kinase gene family [49]. The c-Met receptor is expressed on the surface of epithelial and endothelial cells [51].

HGF is a growth factor for various types of cells: acts as a mitogen for renal epithelial cells, epidermal keratinocytes and melanocytes and others; promote the growth of hepatocytes and hematopoietic cells in culture. The c-Met is present in hematopoietic progenitor cells from human bone marrow and peripheral blood and in the presence of erythropoietin, HGF induces proliferation and differentiation of erythroid progenitors [43].

During embryogenesis HGF and c-Met is crucial, as it was shown that c-Met and HGF play an important role in control of growth, survival and migration of distinct embryonal cells [42]. The c-Met contributes to the development of placental tissue, liver and neuronal precursors and also contributes to the migration and development of muscle tissue by controlling the EMT of myogenic progenitor cells. In animal studies, target mutation HGF or MET, or both genes caused abnormalities that led to embryonic lethality [42].

HGF/c-Met signaling, which is mainly mediated by the RAS–MAPK and PI3K–AKT pathways, affects gene expression and cell cycle progression through the binding of transcription factors, such as the ETS family. Cytoplasmic signaling cascades mediated by PI3K–AKT and the GTPases RAC1 or cell division control protein 42 (CDC42) modulate cell survival and elicit cytoskeletal changes. Signals to the plasma membrane control cell migration and cell adhesion mainly through the RAP1 and RAC1–CDC42 pathways, which affect integrins and cadherins [52].

HGF acts as a pleiotropic factor and cytokine, promoting cell proliferation, survival, motility, scattering, differentiation and morphogenesis. Physiologically, c-Met is responsible for the cell-scattering phenotype, as first demonstrated with MDCK cells treated with HGF. This process involves the disruption of cadherin-based cell-cell contacts and subsequent cell motility [36, 53]. PI3K is an important molecule in HGF-induced mitogenesis, morphogenesis, and chemotaxis [50].

After liver injury, the HGF mRNA is rapidly induced in the lung, spleen and kidney. Therefore, HGF from neighboring cells in the liver and from extrahepatic organs may function as a trigger for liver regeneration by booth paracrine and endocrine mechanisms [44]. *MET* and *HGF* genes were reported to be up-regulated after injury in different epithelial tissues, such as kidney, lung, skeletal muscle, heart, skin, and liver. In the skin, *MET* was shown to be essential for wound repair [54]. In the liver, it was observed that the activation of the HGF/c-Met pathway is essential for DNA synthesis and liver regeneration, but *MET* ablation resulted in impaired proliferation and incomplete liver [55].

In the skin, stem cell populations generate different epidermal cell types during normal turnover and wound repair [52]. The results obtained by Chmlielovic et al. suggest that c-Met is also essential for the generation of the hyperproliferative epithelium in skin wounds, and thus for a fundamental regenerative process in the adult [56]. They reported that both HGF and c-Met were up-regulated in the hyperproliferative epithelium during wound repair in mice, suggesting that HGF and c-Met signal acted in an autocrine manner to promote wound healing. In mutant mice the c-Met was inactivated in the epidermis by the use of a keratin 14 (K14) promoter-driven Cre recombinase. This resulted in the mutation of c-Met in 95% of the epidermal cells. Remarkably, they found that c-Met-mutant keratinocytes were completely unable to re-epithelialize the wounds. Instead, residual keratinocytes that escaped recombination (5%, c-Met–positive cells) closed the wounds, but the wound healing process was delayed. These results demonstrate that the c-Met signaling is essential for skin wound healing. Apparently, no other signaling system is able to compensate for a lack of the c-Met in this process.

**Fig. 1:**
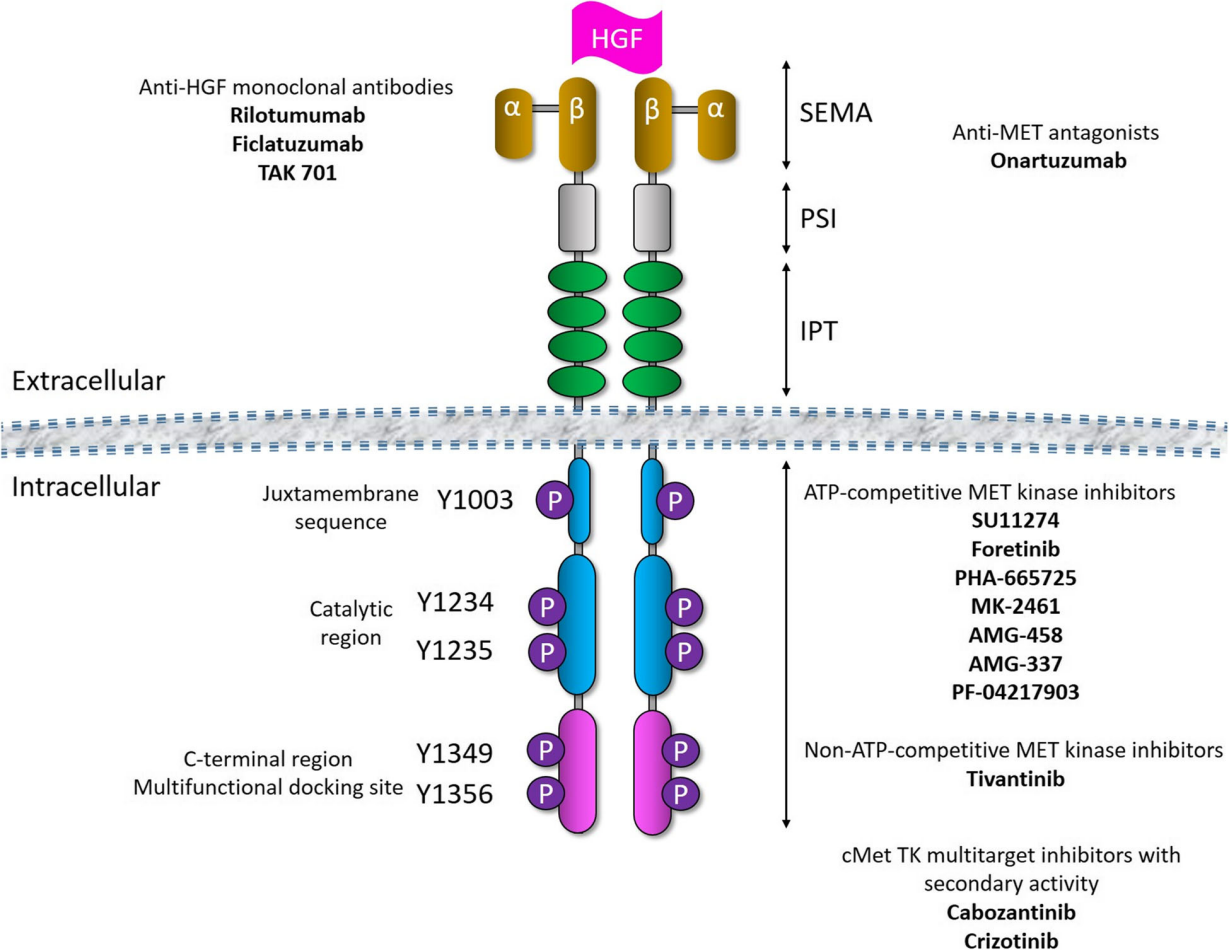
Schematic structure of c-MET protein and the action sites of the inhibitors. Abbreviations: P: phosphate group; PSI - plexins-semaphorins-integrins; IPT - immunoglobulin-plexin-transcription

**Fig. 2:**
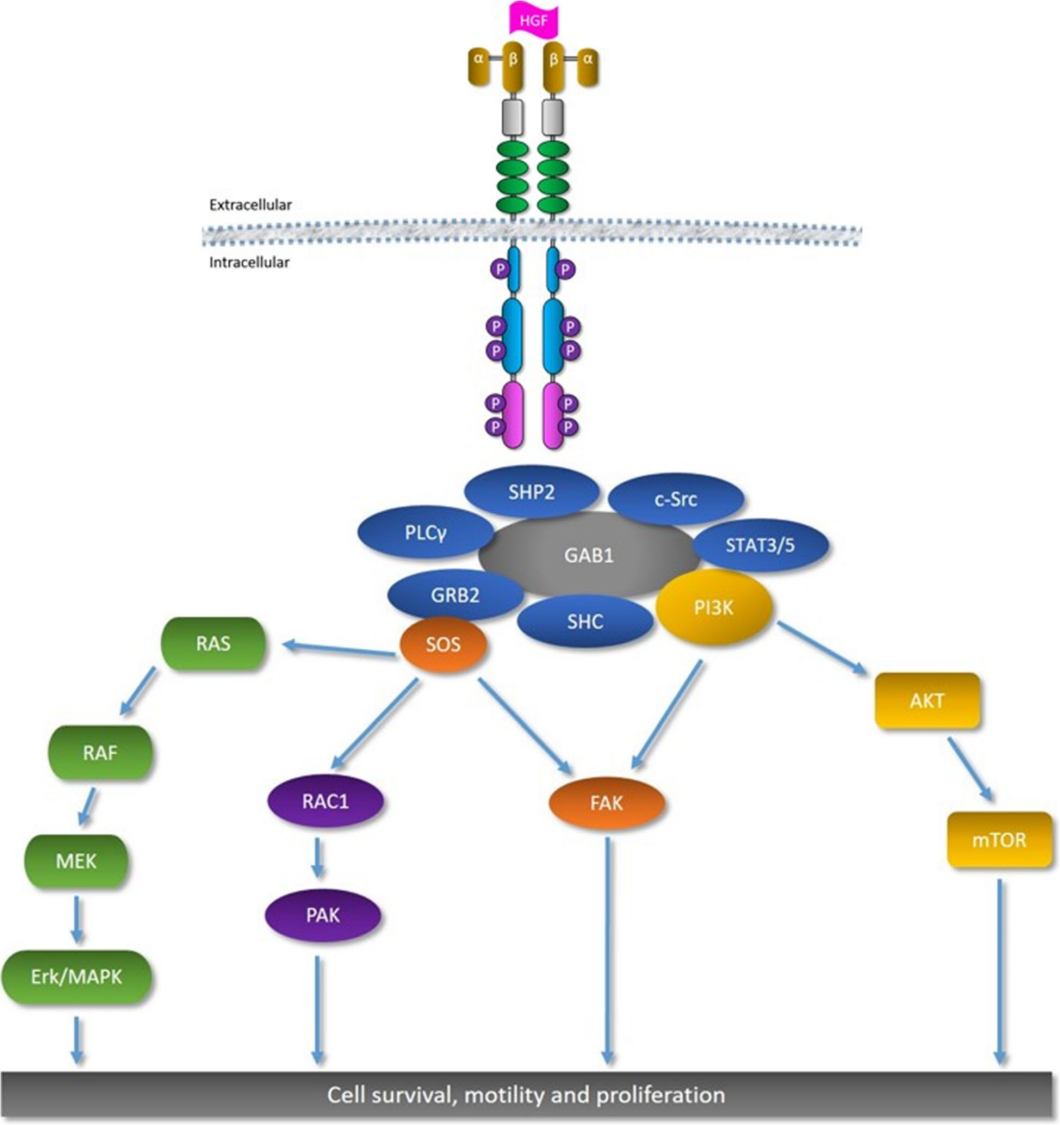
Downstream signaling interactions of the HGF/c-MET signaling pathway. Abbreviations: ERK/MAPK - extracellular signal-regulated kinase/mitogen-activated protein kinases; STAT - signal transducer and activator of transcription factor; GRB2- growth factor receptor-bound protein 2; GAB1, GRB2-associated binding protein 1; PLCy - phospholipase C; PI3K - phosphoinositol 3-kinase; Akt - protein kinase B; mTOR - mammalian target of rapamycin

Ligand-induced c-Met dimerization activates the tyrosine kinase by phosphorylation of tyrosine residues (Tyr1230, Tyr1234 and Tyr1235) in the kinase domain. This initial phosphorylation cascade leads to autophosphorylation of the carboxy-terminal bidentate substrate-binding site (Tyr1349 and Tyr1356) of the c-Met and these residues have been shown as docking sites for downstream signaling molecules [52]. HGF induces dimerization and activation of the c-Met at the plasma membrane. The cytoplasmic tyrosine phosphorylation (P) sites of the c-Met are indicated: Tyr1003 is in the juxtamembrane binding site, Tyr1234 and Tyr1235 are in the active site of the kinase and Tyr1349 and Tyr1356 are in the bidentate docking site (Fig. 1) [52]. Following HGF-mediated dimerization and autophosphorylation of the c-Met receptor, signaling proteins are recruited to the carboxy-terminal docking site, either directly or indirectly through growth factor receptor-bound protein 2 (Grb2) and Grb2-associated binder-1 (Gab1). This leads to the activation of downstream pathways such as Erk/MAPK and Akt/PKB, and it leads to biological responses such as cell proliferation, transformation, survival, migration and angiogenesis (Fig. 2). Signaling proteins directly or indirectly recruited to the phosphorylated docking site include the growth factor receptor-bound protein 2 (Grb2) adaptor, the non-receptor tyrosine kinase Src, Src homology 2 domain-containing (Shc) adaptor protein, the p85 subunit of phosphatidylinositol 3′ kinase (PI3K), phospholipase C γ (PLCγ), tyrosine phosphatase SHP2, Src homology 2-containing inositol 5-phosphatase 1 (SHIP2), signal transducer and activator of transcription 3 (STAT3) and the multisubstrate docking protein Grb2-associated binding protein (Gab1) [57]. The HGF/c-Met pathway mediates downstream signaling through Ras/Raf/MAPK, PI3K/AKT/mTOR, and/or STAT3/5 pathways [58].

### HGF/c-met signaling pathway in metastatic process

The c-Met, and its ligand HGF have been associated with tumor formation and progression to metastasis, with *MET* gene often overexpressed or mutated. Thus, the c-Met has become a major target for cancer therapy and its inhibition is currently being tested in the clinical trials [45]. HGF/c-Met signaling pathway with its downstream effector components (MAPK, STAT, PI3K-AKT cascades and NF-κB) increase cell survival, motility and proliferation [51].

Dysregulation of c-Met correlated with a poor prognosis. Interestingly, abnormal activation of c-Met signaling is implicated in the acquisition of tumorigenic and metastatic phenotypes in tumors. Examinations indicated that c-Met was expressed and activated in melanoma tissues and cell lines. It was shown, that overexpression of c-Met was associated with melanoma growth and metastasis [59]. High expression of the c-Met receptor was detected in metastatic melanoma cell line EGFP-A375iv and in hypermetastatic human melanoma cell line Rel3, which were derived from human melanoma cell line A375. These cell lines are highly tumorigenic and hypermetastatic, which was proven by lung colonization assay. The tumor cells were intravenously injected in SCID mice and all animals had the tumor infiltration in the lungs. It is obvious that the c-Met signaling plays important role in melanoma metastasis [60].

Hypoxia is a key regulator of c-Met, as it induces the expression of the transcription factor hypoxia inducible factor 1 alfa (HIF-1α). The existence of this correlation is supported by preclinical studies in mouse xenograft models, which showed that the therapeutic inhibition of angiogenesis reduces tumor vascularization and causes hypoxia, and therefore may promote c-Met-mediated invasion of malignant cells [50]. The *MET* gene is activated by point mutations in small-cell lung cancer (SCLC) and renal papillary carcinomas. c-Met protein is overexpressed in melanoma and musculoskeletal tumors [61]. Cross-talk between c-Met and EGFR is implicated in tumorigenesis [50].

It has been shown, that many types of tumors express both the ligand - HGF and receptor - c-Met. Not all of them are associated with a poor prognosis or with mutations in the *MET* gene [41]. In melanoma, point mutations N375S, T1010I and R988C, which were associated with NRAS and BRAF mutations, were detected [62]. Activation of c-Met in cancer occurs most often through ligand-dependent autocrine or paracrine mechanisms. In glioblastoma, gastric and head and neck tumors expressing both c-Met and HGF, the mutation of *MET* was found and correlated with poor prognosis in patients. Contrastingly, point mutations in the *MET* gene and correlation with prognosis was not found in the malignant melanoma [41].

### HGF and c-met inhibitors in anticancer therapy

The c-Met is not only a marker linked to metastatic properties but is also a suitable drug target and the molecules which inhibit HGF/c-Met signaling are expected to serve for therapeutic intervention [51]. Several intervention strategies were designed to affect HGF/c-Met signaling: inhibitors of HGF activation, HGF neutralizing antibodies Rilotumumab (AMG102), Ficlatuzumab (AV-299) and TAK701 [61], c-Met antagonists Onartuzumab, CE-355621, selective inhibitors of c-Met kinase activity Tivantinib, AMG-337, AMG-458, Foretinib, PHA-665725, MK-2461, PF-04217903 and SU11274, and nonselective multitarget inhibitors of RTK including c-Met such as Crizotinib and Cabozantinib. ATP binding to the c-Met, inhibiting receptor transactivation and recruitment of the downstream effectors can be achieved also by low molecular weight inhibitors SU11274 and PHA-665752. They block phosphorylation of the c-Met on Tyr1234/1235. PHA-665752 inhibits quite specifically c-Met kinase catalytic activity [63]. Overview of the HGF/c-MET inhibitors currently used in melanoma treatment with references to the in vitro, in vivo and clinical studies is in Table 1.

**Table 1:** List of HGF/c-MET inhibitors currently used in melanoma treatment with references to the in vitro, in vivo and clinical studies

INHIBITOR	Activity	Use in melanoma treatment	Refs
AMG-337	ATP-competitive, highly selective inhibitor of the c-Met receptor	No	[73]
AMG-458	Potent inhibitor of the c-Met, VEGFR-2, and IGF-I signaling pathways	No	[74]
Cabozantinib (XL184, BMS-907351)	Inhibitor of tyrosine kinases including VEGF receptors, c-Met and AXL	In vitro In vivo – clinical trial NCT00940225 NCT01835184	([39, 87, 88], https://clinicaltrials.gov/ct2/show/NCT00940225, https://clinicaltrials.gov/ct2/show/record/NCT01835184)
Crizotinib (PF-02341066)	Potent inhibitor of the c-Met and ALK	In vitro In vivo – clinical trial NCT02223819 (uveal melanoma)	(https://clinicaltrials.gov/ct2/show/NCT02223819, [89])
Foretinib (EXEL-2880)	ATP-competitive inhibitor of the c-Met and VEGFR	In vitro Animal study	[78]
MK-2461	Multi-targeted inhibitor of the c-Met (WT/mutants), c-Met (Y1235D), c-Met (Y1230C), c-Met (N1100)	No	
PF-04217903	Selective ATP-competitive c-Met inhibitor	In vitro	[90]
PHA-665752	Inhibitor of Y1234 and Y1235 in catalytic region of the c-Met	In vitro	[80, 81]
SU11274	Selective inhibitor of Y1234 and Y1235 in catalytic region of the c-Met	In vitro Animal study	[65, 66, 69]
Tivantinib (ARQ 197)	Non-ATP-competitive c-Met inhibitor binding to the dephosphorylated c-Met kinase in vitro	In vitro In vivo – clinical trial NCT00827177	([66], https://clinicaltrials.gov/ct2/show/NCT00827177)

SU11274 was identified as a prototype ATP-competitive small molecule inhibitor of the catalytic activity of c-Met [64]. The expression of c-Met RTK protein was studied in seven melanoma cell lines and the 140-kDa β subunit of c-Met protein was expressed in six of seven melanoma cell lines. The IC_50_ of SU11274 was between 1 and 2.5 μmol/L and apoptosis was observed in five melanoma cell lines that expressed c-Met [65]. It was found that SU11274 as a possible monotherapy significantly reduced size of melanoma in mice. The inhibition of vessel formation by decreased VEGF expression and increased Thrombospondin-1 expression resulted from the c-Met inhibition. This suggested c-Met inhibition as a promising therapeutic option for HGF producing, c-Met TKI sensitive tumors in melanoma patients [66]. SU11274 inhibits HGF-dependent phosphorylation of c-Met as well as HGF-dependent cell proliferation and motility. In human small cell lung cancer cell lines - H69 and H345 which have functional c-Met receptor, SU11274 inhibits the HGF-induced cell growth with the IC_50_ of 3.4 μM and 6.5 μM, respectively. SU11274 induces G1 cell cycle arrest with cells in G1 phase increased from 42.4% to 70.6% at 5 μM, and induces caspase-dependent apoptosis by 24% at 1 μM. SU11274 inhibits cell viability in c-Met-expressing non-small cell lung cancer (NSCLC) cells with IC_50_ values of 0.8–4.4 μM, and abrogates HGF-induced phosphorylation of c-Met and its downstream signaling [64, 67].

The inhibitor SU11274 specifically decreased the phosphotyrosine signal at the focal adhesion sites in multiple myeloma cells, which was accompanied by a decrease in cell proliferation as well as an increase in number of apoptotic cells. Moreover, the SU11274 significantly reduced the in vitro migratory capacity of myeloma cells and treatment by the SU11274 decreased primary tumor growth and the capacity for liver colony formation in SCID mice [68].

High level of the c-Met receptor was confirmed in human melanoma cells M14, M4Beu, A375 and Rel3, and the IC_50_ for c-Met inhibitor SU11274 was 4–5 μM [69].Contrary to the expected effect of HGF/c-Met inhibitor, in our experiments with the hypermetastatic and highly tumorigenic variant of human melanoma cell line A375 designated as Rel3 we have shown that SU11274 enriched for the melanoma-initiating cells in vivo. In the adherent cell cultures treated with the inhibitor SU11274 we have observed significantly reduced number of cells, change in the cell morphology, reduction in the proliferation and increased tumorigenicity. This inhibitor substantially decreased the number of cells in adherent and spheroid cultures as well, nevertheless it has increased their tumorigenic potential as determined by higher frequency of tumor-initiating cells in vivo. Results show that the SU11274 treatment was not associated with any significant alteration in the expression of stem cell markers, but the inhibitor stimulated higher level of pluripotent markers. We described that the SU11274-treated melanoma cells exhibited higher ATP content and lactate release indicative of increased glycolysis. Based on these data we suggest that the SU11274 altered bioenergetic state of the cells. Indeed, pharmacological intervention with a glycolytic inhibitor dichloroacetate has significantly reduced SU11274-promoted increase in melanoma-initiating cells and decreased their tumorigenicity [69]. In the adherent Rel3 cells treated with SU11274 we noticed increased expression of MUSASHI-1, which is considered as a marker of cancer stem cells (unpublished data). However, we detected a decrease in the expression of Nestin, a marker of proliferation and migration. In Rel3 spheroid cells treated with the SU11274 there is also increased expression MUSASHI-1 along with an increase in Nestin, which can be also related to the increased cell motility, invasiveness and malignancy. The SU11274 treatment upregulated almost 2-fold several other pluripotency markers (Oct3/4, Nanog, AFP and Gata4) in the treated cells (unpublished data). It also increased activity of RSK1/2/3 kinase based on the phosphotyrosine array analysis [69].

Crizotinib (PF-02341066) as a potent inhibitor of c-Met and ALK received approval for the treatment of patients with locally advanced or metastatic NSCLC that is ALK-positive in 2013 [70]. Crizotinib inhibits HGF-stimulated human NCI-H441 lung carcinoma cell migration and invasion with IC_50_ of 11 nM and 6.1 nM, respectively. It was identified as a potent, orally bioavailable, ATP-competitive small-molecule inhibitor of the catalytic activity of c-Met kinase. Crizotonib was selective for the c-Met (and anaplastic lymphoma kinase) compared with a panel of > 120 diverse tyrosine and serine-threonine kinases. It potently inhibited c-Met phosphorylation and c-Met-dependent proliferation, migration, or invasion of human tumor cells in vitro (IC_50_ values, 5–20 nmol/L). In addition, crizotinib potently inhibited HGF-stimulated endothelial cell survival or invasion and serum-stimulated tubulogenesis in vitro, suggesting that this agent also exhibits antiangiogenic properties [71, 72]. We have shown, that the IC_50_ for crizotinib ranged 1.25–3 μM in standard adherent cultures of melanoma cell lines M14, M4Beu, A375 and Rel3 [69]. Inhibitor crizotinib is being administered to patients with uveal melanoma who are at high risk of recurrence in a phase II clinical trial, but the patient recruitment is ongoing and no results have been published (https://clinicaltrials.gov/ct2/show/NCT02223819).

There are several other inhibitors available for the inhibition of the c-Met signaling (Fig. 1). AMG-337 is a small molecule, ATP-competitive, highly selective inhibitor of the c-MET receptor. AMG-337 inhibits c-MET phosphorylation and downstream signaling through the PI3K and MAPK pathways in gastric cancer cell lines SNU-638 and IM-95 [73].

AMG-458 is a potent inhibitor of the c-Met, VEGFR-2, and IGFR1 receptor signaling pathways, inhibitor with radiosensitizing effects. AMG-458 significantly inhibited tumor growth of the U-87 MG human glioblastoma xenografts and of NIH-3 T3/TPR-MET tumors with constitutive activation of c-Met [74]. The combination of radiation therapy and AMG-458 treatment was found to synergistically increase apoptosis in the H441 cell line but not in lung adenocarcinoma cells A549. AMG-458 significantly enhances the radiosensitivity of H441 [75].

Foretinib (EXEL-2880) is an ATP-competitive multi-kinase inhibitor inhibitor of the c-Met receptor and VEGFR [76]. Orthotopic tumors treated by foretinib exhibited decreased lymphangiogenesis, angiogenesis and cell proliferation. It was detected that the expression of LYVE-1 (lymphatic vessel endothelial hyaluronan receptor 1), CD31 (platelet endothelial cell adhesion molecule) and Ki-67 were reduced. The mean of lymph vessel density in the tumors and also the percentage of lymph vessel area was reduced by Foretinib. In addition, the mean of blood vessel density and the percentage of blood vessel area in tumors were suppressed by 70–80%. Foretinib simultaneously inhibits cancer cells and lymphatic endothelial cells to reduce pancreatic tumor growth in vivo*,* and this data demonstrated for the first time that this inhibitor suppresses angiogenesis and lymphangiogenesis by blocking VEGFR-2/3 and TIE-2 signaling [77]. Foretinib (EXEL-2880) also inhibits HGF-driven migration and invasion of murine B16F10 melanoma cells (value IC_50_ of 21 nmol/L). Single-dose oral administration of EXEL-2880 resulted in prolonged inhibition of phosphorylation of constitutively phosphorylated Met in B16F10 solid tumors as well as ligand HGF-stimulated phosphorylation of Met in whole liver [78].

Cabozantinib (XL184, BMS-907351) is an inhibitor of tyrosine kinases including VEGF receptors, c-Met and AXL. Cabozantinib has clinical activity in patients with metastatic melanoma, including uveal melanoma. The data from the clinical trial point out benefits of cabozantinib on both soft tissue and bone lesions in patients with metastatic melanoma. The data indicate that targeting of the VEGFR, c-Met and AXL pathways with cabozantinib tends to improve outcomes in patients with metastatic melanoma. Treatment with cabozantinib was associated with encouraging progression-free survival, overall survival and reduction in the size of measurable target lesions was observed in the majority of patients with uveal, cutaneous, and mucosal melanoma [39]. XL184 is useful at low concentration (0.1–0.5 μM) to induce marked inhibition of constitutive and inducible c-Met phosphorylation and its consecutive downstream signaling in malignant peripheral nerve sheath tumor cells. In these cells, it inhibited HGF-induced cell migration and invasion, xenograft growth and metastasis in SCID mice. XL184 also induced marked inhibition of Met and VEGFR2 phosphorylation in cytokine-stimulated human umbilical vein endothelial cells [79].

PHA-665752 is a potent, selective and ATP-competitive c-Met inhibitor, PHA-665752 inhibited c-Met tyrosine phosphorylation at the activation loop (pY1230/34/35), multifunctional docking site (pY1349), and the juxtamembrane domain (pY1003) at 0.1 μM [80]. NRAS-mutated melanoma cell lines (SB2 and SK-Mel-2) migrate efficiently toward HGF but this process is completely inhibited by PHA-665752, and treatment with 50–100 nM PHA-665752 inhibited phosphorylation of Akt. It shows an association of mutated NRAS with increased HGF-dependent activation of c-Met and with enhanced sensitivity to c-Met inhibition [81]. Effective inhibition of c-Met, p-AKT, and p-ERK was achieved by dual inhibition with the BRAF inhibitor (PLX4032) and c-Met inhibitor (PHA665752). The effect was investigated on two thyroid cancer cell lines, 8505C (anaplastic thyroid cancer) and BCPAP (papillary thyroid cancer) and dual inhibition of BRAF and c-Met led to sustained treatment response. Similar results were confirmed by in vivo study on orthotopic xenograft mouse model [82].

MK-2461 is another potent, ATP-competitive multi-targeted inhibitor of the c-Met (WT/mutants): c-Met (Y1235D), c-Met (Y1230C) and c-Met (N1100). In tumor cells, MK-2461 effectively restrained constitutive or ligand-induced phosphorylation of the juxtamembrane domain and COOH-terminal docking site of c-Met. In the cell culture, MK-2461 inhibited HGF/c-Met–dependent mitogenesis, migration, cell scatter, and tubulogenesis [83].

PF-04217903 is a selective ATP-competitive c-Met inhibitor with IC_50_ of 4.8 nM in A549 cell line, sensitive to oncogenic mutations (no activity to Y1230C mutant). PF-04217903 in association with sunitinib radically inhibits endothelial cells, but not the tumor cells B16F1, Tib6, EL4, and LLC. It heftily inhibits c-Met-driven processes like cell growth, motility, invasion, and morphology of a diversity of tumor cells [84].

Tivantinib (ARQ 197) is a staurosporine derivative and it represents the first non-ATP-competitive c-Met inhibitor that binds to the dephosphorylated c-Met kinase in vitro. It is clinically tested as a highly selective c-Met inhibitor. In all cell models analyzed, tivantinib did not inhibit HGF-dependent or HGF-independent c-Met tyrosine autophosphorylation [85].

There are several antibodies targeting HGF/c-Met signaling. Rilotumumab (AMG-102) binds the HGF light chain, locking out the HGF/c-Met binding. Ficlatuzumab (AV-299) is a humanized monoclonal anti-HGF IgG1 antibody that enchains to HGF, thereby inhibiting the HGF/c-Met interplay. TAK-701 is a humanized anti-HGF monoclonal antibody that was found to outperform gefitinib defiance in *EGFR*-mutated human NSCLC cell lines both in vitro and in xenograft mouse models [61].

In summary, current strategies in oncology shift towards the targeted treatment exploiting identification of crucial signaling pathways for metastatic spread of tumor cells. The involvement of the HGF/c-Met signaling in solid tumors, including melanoma, prompted development of new drugs, which have already brought benefit in clinical setting. In the treatment of NSLSC, the crizotinib doubled the survival of patients [70]. We suppose that it might bring benefit for the melanoma patients in future as well. Disrupting the HGF/c-Met signaling may interfere with tumor cell scattering thus affecting the metastasis dissemination. Its physiological function, which is restricted in adults, brings potential tumor-specific effect. Blocking the HGF/c-Met signaling and reducing phosphorylation in this pathway reduces phosphorylation downstream of the receptor. About 50% of patients with melanoma have BRAF mutations and 15–20% of NRAS mutations leading to the constitutive activation of the MAPK signaling pathway [2] and Chattopadhyay et al. suggested that decreased c-Met activity in melanoma cells could be a useful therapeutic strategy [81]. However, it must be carefully evaluated as there might be unwanted side effects of the treatment. We have observed increased tumorigenicity upon interference with the HGF/c-Met signaling with the SU11274. The precise mechanism may involve multiple processes including compensatory receptor crosstalk and it is to be further investigated. It has been shown that the crizotinib induces alterations in the secretome of the melanoma cells contributing to the emergence and expansion of the resistant subpopulations [86]. It remains to be further investigated, whether it is possible to avoid blunting of the inhibitory action by other types of inhibitors, by their combination or by combination of the diverse therapeutic modalities to efficiently limit propagation of tumor cells. Other potential risk of the HGF/c-Met inhibition by the systemic treatment is in affecting the tissue regeneration and damage repair processes in patients, although we did not observe any side effects on our mouse models upon SU11274 administration.

Taken together, many results of the in vitro experiments and clinical studies show that the most appropriate treatment is a combination of different inhibitors chosen based on the molecular properties of tumor cells. Targeted therapies have a potential of achieving control over the metastatic disease with limited toxicity and controlling the disease with long-term survival of patients.

## Conclusions

In this review we describe the major factors affecting metastatic process with a focus on the malignant melanoma. As there are many attempts to find targeted therapy to control the metastatic dissemination, we focus here the signaling pathway c-Met/HGF, which is involved in many aspects of tumorigenesis including cell dissemination and metastasis. We also summarized the outcomes of the recent studies using specific inhibitors of this pathway in an attempt to limit metastatic spread, tumor growth with a focus on limiting melanoma proliferation and tumorigenicity. Detailed understanding of the inhibitory action exerted by targeted RTK inhibitors including those affecting the HGF/c-Met signaling is critical for the durable antitumor responses.

## Abbreviations

ATP: Adenosine triphosphate
c-Met (HGFR): Hepatocyte growth factor receptor
EGFR: Epidermal growth factor receptor
EMT: Epithelial – mesenchymal transition
HGF: Hepatocyte growth factor
MAPK: Mitogen-activated protein kinase
MET: Mesenchymal-to-epithelial transition
MM: Malignant melanoma
NF-κB: Nuclear factor-κB
NK cells: Natural killer cells
PDGFR: Platelet-derived growth factor receptor
PI3K: Phosphatidylinositol-3 kinase
PTKs: Protein tyrosine kinases
Rel3 (EGFP-A375/Rel3): Hypermetastatic human malignant melanoma cell line derived from EGFP-A375
RTKs: Receptor tyrosine kinases
TGF-β: Transforming growth factor-Beta
